# Chemical Synthesis and Insecticidal Activity Research Based on α-Conotoxins

**DOI:** 10.3390/molecules29122846

**Published:** 2024-06-14

**Authors:** Chengzhang Lin, Hailong Qin, Yanling Liao, Jiao Chen, Bingmiao Gao

**Affiliations:** Engineering Research Center of Tropical Medicine Innovation and Transformation of Ministry of Education, International Joint Research Center of Human-Machine Intelligent Collaborative for Tumor Precision Diagnosis and Treatment of Hainan Province, Hainan Key Laboratory for Research and Development of Tropical Herbs, School of Pharmacy, Hainan Medical University, Haikou 571199, China; linchengzhang@hainmc.edu.cn (C.L.); m202375869@hust.edu.cn (H.Q.); liaoyanling@hainmc.edu.cn (Y.L.)

**Keywords:** *Conus quercinus*, α-conotoxins, insecticidal activity, mechanism prediction

## Abstract

The escalating resistance of agricultural pests to chemical insecticides necessitates the development of novel, efficient, and safe biological insecticides. *Conus quercinus*, a vermivorous cone snail, yields a crude venom rich in peptides for marine worm predation. This study screened six α-conotoxins with insecticidal potential from a previously constructed transcriptome database of *C. quercinus*, characterized by two disulfide bonds. These conotoxins were derived via solid-phase peptide synthesis (SPPS) and folded using two-step iodine oxidation for further insecticidal activity validation, such as CCK-8 assay and insect bioassay. The final results confirmed the insecticidal activities of the six α-conotoxins, with Qc1.15 and Qc1.18 exhibiting high insecticidal activity. In addition, structural analysis via homology modeling and functional insights from molecular docking offer a preliminary look into their potential insecticidal mechanisms. In summary, this study provides essential references and foundations for developing novel insecticides.

## 1. Introduction

Cone snails are a class of carnivorous mollusks predominantly found in tropical marine environments [1,2,3]. Taxonomically, they are classified under the phylum Mollusca, the class Gastropoda, subclass Neogastropoda, and family Conusidae [4,5]. One notable species within this family is *C*. *quercinus*, first identified and named by the botanist John Lightfoot in 1786 [6]. The habitat of *C*. *quercinus* is expansive, encompassing the South China Sea, the Red Sea, the Indian Ocean, and the Pacific Ocean. Cone snails exhibit various predatory behaviors, classifying them into distinct groups: piscivores, fish hunters; vermivores, worm hunters; and molluscivores, which prey on other mollusks [7,8]. The *C. quercinus* species is a vermivore. Upon detecting its prey, the cone snail employs a rapid and precise hunting mechanism. It swiftly ejects its venom-filled, needle-like tongue from its elongated “kiss” into the prey, paralyzing it within seconds. The venom, pivotal to this predatory strategy, is a complex cocktail of numerous toxic peptides, collectively termed conotoxins.

Conotoxins are a class of neurotoxic peptides, typically ranging from 7 to 46 amino acid residues in length with a high cysteine content [9]. Based on the number and arrangement of cysteine residues within their sequences, conotoxins are categorized into distinct superfamilies: A, O, M, P, I, and T. Each superfamily is further divisible into subfamilies [10,11,12]. For instance, α-conotoxins are a subset of conotoxins belonging to the A-superfamily and are defined by their cysteine framework (CC-C-C). These peptides are predominantly known for interacting with the nicotinic acetylcholine receptor (nAChR) [13]. The unique structural attributes of α-conotoxins, potent biological activity, and high target specificity render them highly valuable in drug development [14]. Of particular interest is their application as potential insecticides, where their precision in targeting nAChRs could offer a novel and effective approach to pest control [15,16,17]. It is estimated that an individual cone snail can synthesize 50–200 distinct conopeptides, with a low likelihood of sequence similarity among toxins produced by different cone snails [18]. Given the approximately 800 recognized species of cone snails worldwide, the global conotoxin diversity could exceed 80,000 unique variants [19,20]. However, less than 0.1% of these conotoxins have been carefully studied and published so far.

Acquiring small molecular active peptides, such as conotoxins, is primarily facilitated through natural extraction, chemical synthesis, and recombinant biosynthesis. The natural extraction method involves directly isolating venom from the venom ducts of live cone snails. However, this approach is limited by the low toxin yields and the complexity of the peptide mixtures, necessitating intricate purification processes. Consequently, it is predominantly utilized for initial research and characterizing novel cone snail species. Recombinant biosynthesis leverages gene editing technologies to produce specific conotoxins in heterologous expression systems, such as *Escherichia coli*, *Pichia pastoris*, and baculovirus-insect cells. Despite this method’s potential, challenges arise with isolating and purifying insoluble inclusion bodies, correctly folding them into a native spatial structure, and screening post-intracellular expression.

In comparison, the chemical synthesis method is ideal for short peptides such as conotoxins with a high demand and short sequences. This technique encompasses solid-phase peptide synthesis (SPPS) and the strategic formation of disulfide bonds. Furthermore, it allows for modification of the peptide’s basic framework to engineer conotoxins with enhanced value and utility [21]. Numerous studies have underscored the substantial potential of conotoxins as a novel class of insecticides [22,23,24]. With the escalating frequency of large-scale insect infestations in recent years, agricultural produce has suffered considerable damage globally, leading to significant economic repercussions. Consequently, proactive measures to mitigate the impact of insect pests on crops are imperative.

Traditional chemical pesticides, including organophosphorus, organochlorine, and nicotine compounds, are known to leave residues on agricultural products, contributing significantly to environmental pollution [25,26]. In contrast, peptide derivatives have demonstrated a novel, efficacious, and safe profile that surpasses conventional agrochemicals [27,28,29]. Among the spectrum of venom-derived biological insecticides, conotoxins have increasingly captured attention for their superior insecticidal properties [30]. As small molecular weight peptides, conotoxins offer potent activity, facile degradation, and non-volatility benefits, positioning them as promising candidates in developing environmentally friendly, green biological insecticides [31].

In our preceding study [13], we employed high-throughput transcriptome sequencing to analyze the venom of *C. quercinus*, culminating in assembling an extensive library encompassing 133 conotoxin transcripts. Using the established sequence characteristics of A-superfamily conotoxins known for their potent insecticidal properties, we applied stringent selection criteria based on the cysteine framework (CC-C-C) and a 12–20 amino acid sequence length [32]. This stringent filtering process identified six conotoxins with potential insecticidal properties from our comprehensive transcriptome library. This study chemically synthesized these six α-conotoxins via SPPS [17]. Additionally, we devised an efficient oxidation and folding strategy specifically for these hydrophilic peptides. The synthesized conotoxins were then subjected to a series of insecticidal activity assays. These investigations aimed to contribute to the existing body of knowledge and facilitate the development of novel, high-efficiency biological insecticides leveraging the unique properties of conotoxins.

## 2. Results

### 2.1. Screening of α-Conotoxins from C. quercinus

Six α-conotoxins were screened from the transcriptome of *C*. *quercinus,* with 17 residues in the length of amino acid sequences (Table 1). Residues with similar properties are highlighted with the same color.

### 2.2. Synthesis of Linear Conopeptides

**Figure 1:**
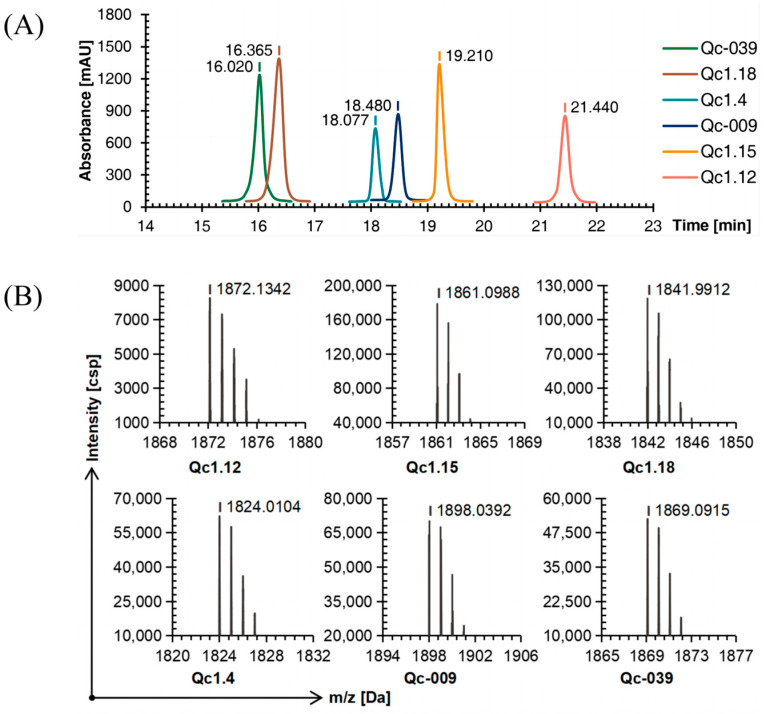
RP-HPLC (**A**) and TOF-MS (**B**) results of six linear conopeptides.

Six linear conopeptides were synthesized using the SPPS method, and the purity was above 95% as determined by C18 reversed-phase high-performance liquid chromatography (RP-HPLC, Figure 1A). Time of flight mass spectrometry (TOF-MS) further verified the obtained linear conopeptide. After analysis, the mass-to-charge ratio of each conotoxin’s linear conopeptide molecular ion peak was consistent with the theoretical molecular mass, indicating that the peptide synthesis was successful, as shown in Figure 1B.

### 2.3. Optimization of Oxidative Folding Conditions

The linear conopeptide Qc1.15, with the highest peak height under the same conditions, was selected as the carrier. Three groups of comparative experiments were completed by changing the use of methanol at various steps.

**Figure 2:**
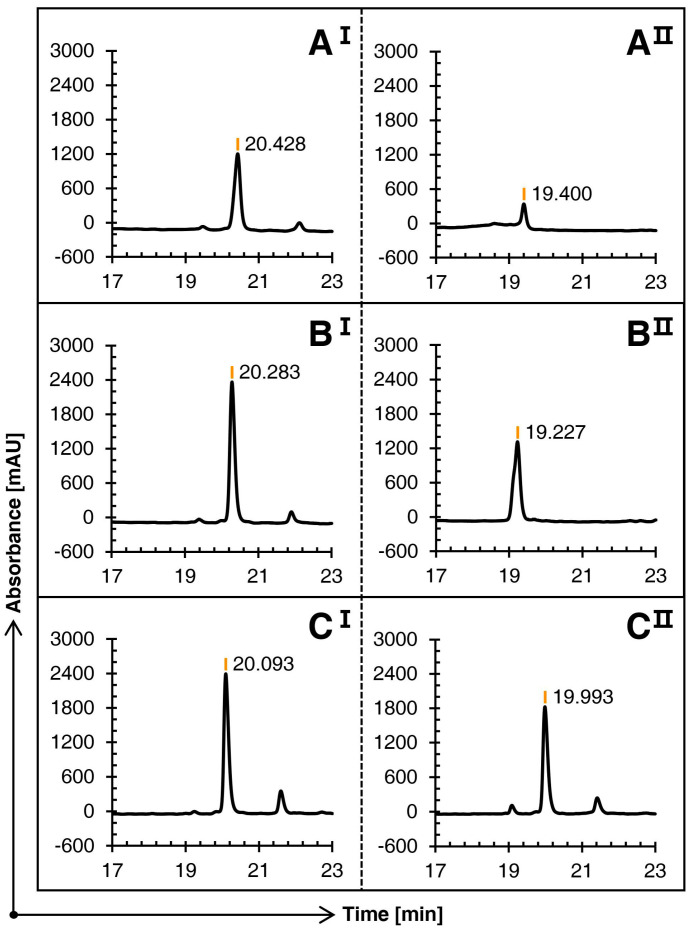
Comparison of the RP-HPLC results of three schemes (**A**), (**B**)**,** and (**C**) of conopeptide Qc1.15. **I** and **II** represent the first and second steps of oxidation folding, respectively.

Figure 2 illustrates that the three schemes can oxidize the first pair of free cysteines into bonds in the first step of oxidation, with no significant difference in their main peak times. However, in contrast, since Schemes B and C avoid the large amount of methanol in the first step of oxidation, the solubility is greatly improved, and the main peak height is superior.

The three schemes showed significant differences in the second oxidation stage. The TOF-MS revealed that the mass-to-charge ratio at the main peak of Schemes A and B was consistent with the theoretical molecular weight; that is, conotoxin Qc1.15 with two pairs of disulfide bonds was formed. The RP-HPLC result of the second oxidation stage of Scheme C shows that it almost stays in the first oxidation stage and fails to continue to catalyze the second pair of cysteines with the acetamidomethyl (Acm) protection group.

### 2.4. Oxidative Folding

**Figure 3:**
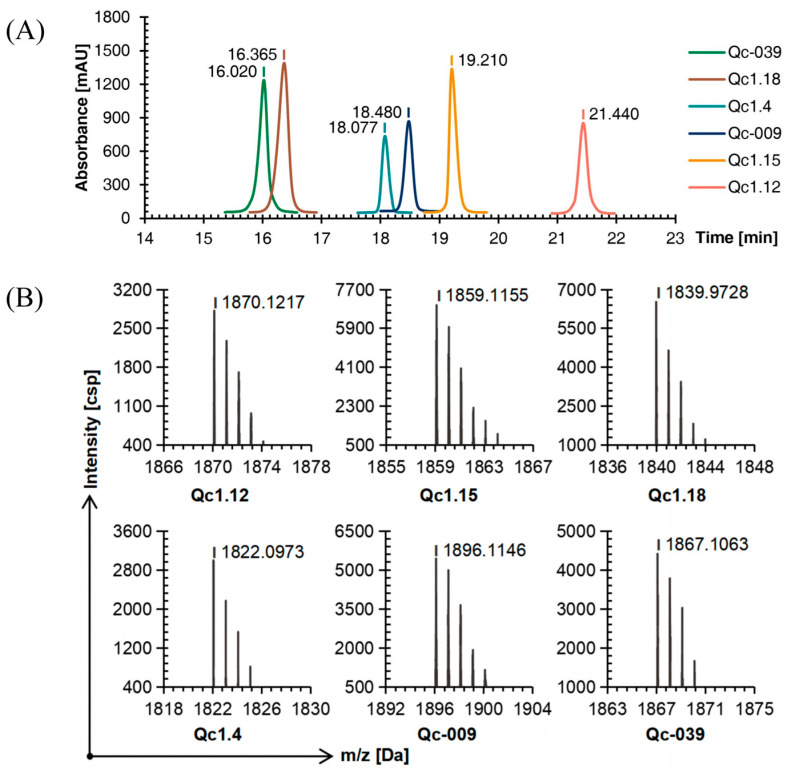
RP-HPLC (**A**) and TOF-MS (**B**) results of six linear conopeptides after forming the first disulfide bond.

Compared with linear conopeptides, the retention time of Qc1.4, Qc1.18, and Qc-039 after oxidation was shortened by about 0.5 min, while those of Qc1.12, Qc1.15, and Qc-009 were prolonged by about 1.5 min, with a similar order of peaks (Figure 3A).

The TOF-MS analysis showed that after the first oxidation step, the mass-to-charge ratio of each conopeptide was about 2 Da different from the molecular weight of the linear conopeptide. This observation proved that two mercapto hydrogens were removed to form a disulfide bond and the first folding was completed (Figure 3B).

**Figure 4:**
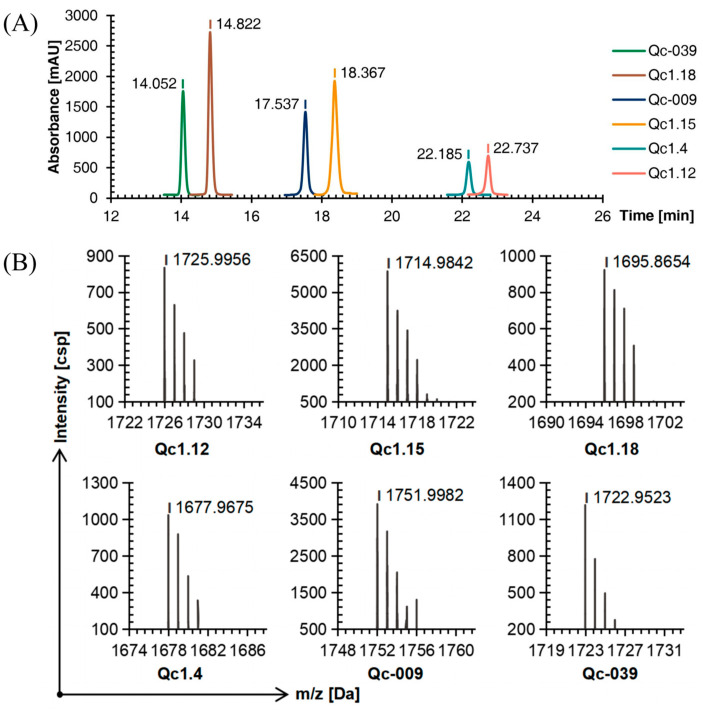
RP-HPLC (**A**) and TOF-MS (**B**) results of six linear conopeptides after forming the second disulfide bond.

As detected by C18 RP-HPLC, the purity of the six conotoxins was more than 95%. Comparing Figure 1A, Figure 3A, and Figure 4A, the peak order of each α-conotoxin barely changed with the increase in disulfide bonds, while the retention time was more dispersed.

The TOF-MS showed that the mass-to-charge ratio of each conopeptide after the second oxidation step was about 144 Da, different from the molecular weight after the first oxidation step and equal to the mass of the two Acm groups. The molecular weight difference between the conopeptide oxidized by the second step and the linear conopeptide was about 146 Da. These deductions also proved that the two protective groups were removed to form two disulfide bonds, and the conotoxin with a natural structure was successfully synthesized (Figure 4B). Finally, the yields of six conopeptides from SPPS to oxidative folding into conotoxins with two disulfide bonds were Qc1.12: 24.78%, Qc1.15: 32.82%, Qc1.18: 49.23%, Qc1.4: 28.72%, Qc-009: 27.47%, and Qc-039: 37.95%.

### 2.5. Cytotoxic Activity of α-Conotoxins from C. quercinus

**Figure 5:**
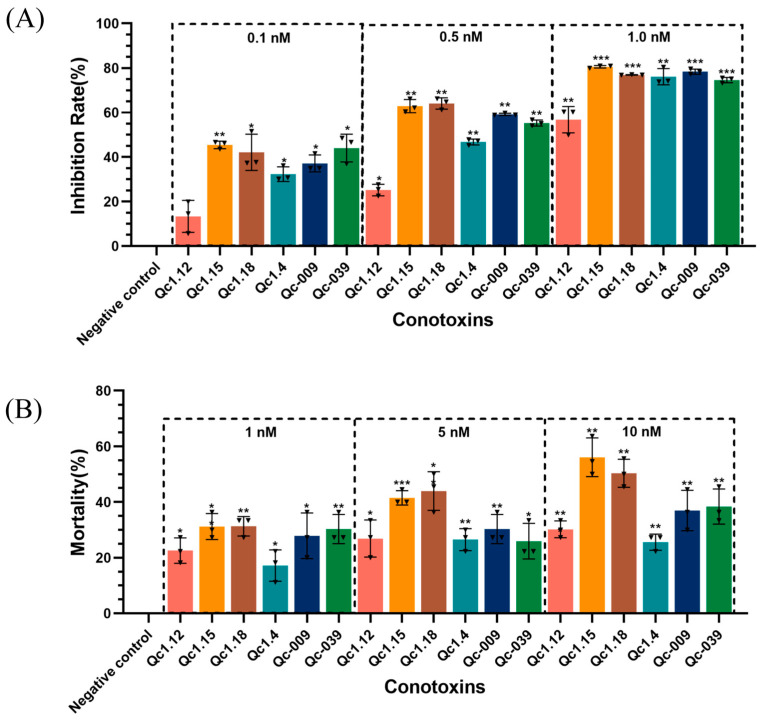
(**A**) Inhibitory effects of α-conotoxins on Sf9 cell growth. (**B**) Insecticidal effects of α-conotoxins on *T. molitor*. Significance (compared with the negative control): * *p* < 0.01, ** *p* < 0.001, *** *p* < 0.0001.

As shown in Figure 5A, the inhibition rate of the experimental group was higher than that of the negative control group, proving that these conotoxins were toxic to *Spodoptera frugiperda* (Sf9) cells.

After calculation, the IC_50_ of the six α-conotoxins on Sf9 cells ranged from 0.15 to 0.86 nM (Qc1.12, 0.86 nM; Qc1.15, 0.15 nM; Qc1.18, 0.19 nM; Qc1.4, 0.46 nM; Qc-009, 0.31 nM; Qc-039, 0.24 nM). Here, Qc1.12 had the lowest inhibitory effect on Sf9 cells, while Qc1.15, Qc1.18, and Qc-039 had the highest inhibitory effects. Specifically, the inhibitory effect of Qc1.15 was the most effective, and its IC_50_ was as low as 0.15 nM, presenting similar inhibitory effects as a known α-conotoxin ImI from *C*. *imperialis* (IC_50_: 0.13 nM) [22]. This α-conotoxin Qc1.15 showed a favorable peak height, folding product purity, and insect individual toxicity under the same conditions.

### 2.6. Insect Toxicity Test of α-Conotoxins from C. quercinus

Figure 5B shows the relationship between six α-conotoxins and the mortality of *Tenebrio molitor* at a given concentration. In general, the mortality of *T*. *molitor* positively correlated with the dose, and there was a significant difference between the negative control group. Still, different conotoxins also had different dose relationships. Among them, Qc1.15 and Qc1.18 had favorable effects at low concentrations, and the mortality gradually increased with increasing dosage. However, the average mortality of Qc1.4 in the medium- and high-concentration groups did not change significantly. At 10 nM, the average mortality of *T*. *molitor* in the Qc1.15 and Qc1.18 groups exceeded 50%. Their median lethal doses were calculated as 7.47 and 9.82 nM, respectively; they were lower than the known LC_50_ value of α-conotoxin ImI (15.0 nM) [22], indicating significant insecticidal activity.

### 2.7. Electrostatic Surface

**Figure 6:**
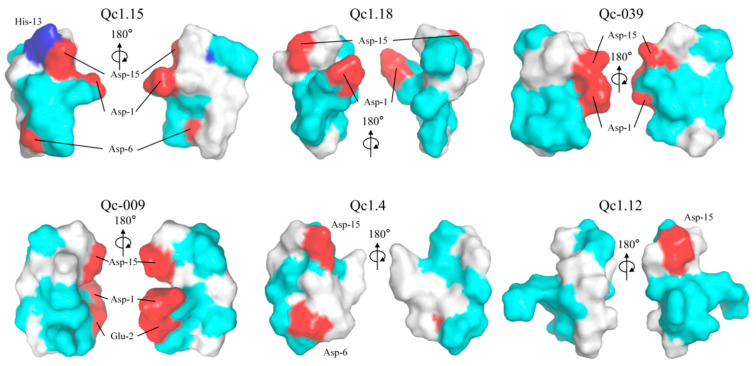
Electrostatic surface potential representations of α-conotoxins Qc1.15, Qc1.18, Qc-039, Qc-009, Qc1.4, and Qc1.12. Some selected residues are labeled. Regions of the surface with basic, acidic, polar neutral, and hydrophobic residues are colored blue, red, cyan, and white, respectively.

These α-conotoxins were examined for their 3D structures by establishing electric potential figures, the homology modeling scores of these α-conotoxins are shown in Table S1. Qc1.15 contains three acidic residues (Asp-1, Asp-6, and Asp-15) and a basic residue (His-13) absent in the other five α-conotoxins (Figure 6). Qc1.18, Qc-039, and Qc1.4 have one less acidic residue than Qc1.15, while Qc1.12 only has one acidic residue (Figure 6). However, the distribution of these acidic residues in spatial structure is inconsistent. The acidic residues of Qc1.15, Qc1.18, and Qc1.4 are relatively separated, while those of Qc-039 and Qc-009 are relatively close.

### 2.8. Predicting the Binding Mode of α-Conotoxins at nAChR

**Figure 7:**
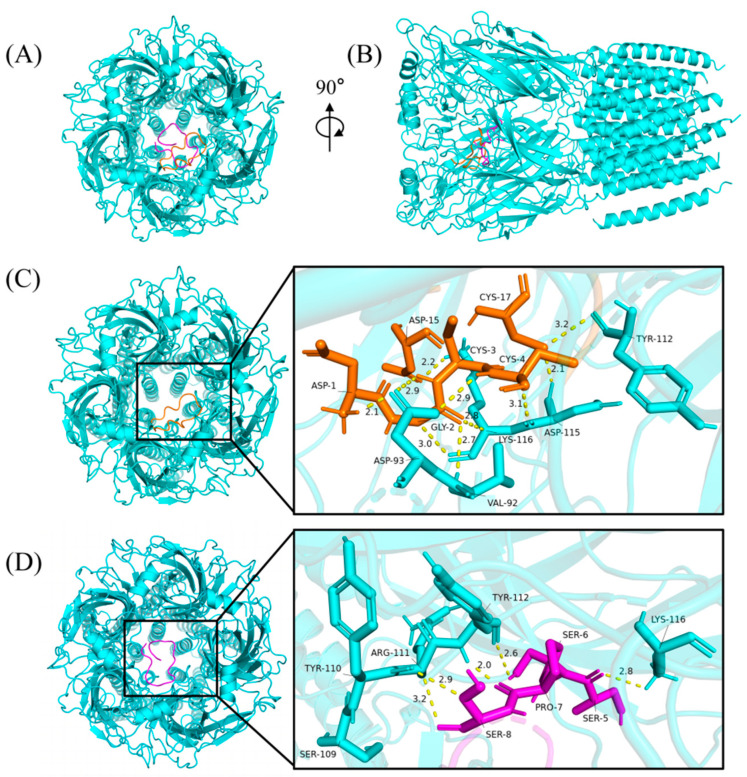
The binding modes of α-conotoxins at nAChR. The Qc1.15, Qc1.18, and nAChR were colored in orange, magenta, and cyan, respectively, from the top (**A**) and side (**B**). The yellow dashed lines show the hydrogen bonds with the distance (Angstroms: Å). The binding modes and magnified images of nAChR/Qc1.15 (**C**) and nAChR/Qc1.18 (**D**), respectively.

The cytotoxicity assays and insect bioassay showed that Qc1.15 and Qc1.18 had high insecticidal activity. Therefore, molecular docking was employed to speculate the interactions of Qc1.15 and Qc1.18 with nAChR of *Alvinella pompejana*. As shown in Figure 7A,B, two α-conotoxins with high performance in cytotoxicity assays and insect bioassay can bind to the central pore of nAChR at various angles and directions, respectively. The docking result showed that ten hydrogen bonds were formed by the central pore residues of the receptor with Qc1.15 (Figure 7C). The binding mode of nAChR/Qc1.18 was highly similar to that bound by Qc1.15, with five hydrogen bonds (Figure 7D). Note that the residues Tyr-112 and Lys-116 of the receptor form hydrogen bonds with Qc1.15 and Qc1.18, where the residues Lys-116 form three hydrogen bonds with the three Qc1.15 amino acid residues. These residues may be crucial in the interaction between nAChR and α-conotoxin entering receptor pores.

## 3. Discussion

For decades, the research on the activity of marine peptides has focused on analgesia, anti-cancer, anti-epilepsy, and other significant diseases, such as conotoxin MVIIA, which has been approved by the FDA as advanced cancer analgesics [33,34,35]. However, most of the peptide toxins secreted by marine organisms are used to prey on arthropods, such as worms and shrimp. It is speculated that their venom can act on insect-specific targets [36,37].

Cone snails constitute the largest single genus of living marine invertebrates and are composed of various predators [38]. The venom gland of cone snails can secrete many unique neurotoxic peptides, usually called conopeptides or conotoxins. Most conotoxins are rich in disulfide bonds and have various pharmacological activities. Each cone snail species usually has 100–200 conotoxins as potential pharmacological targets [39].

Studies on mitochondrial genes found that the venom composition of cone snail species depends on food type and dietary range [40,41]. *C*. *quercinus* is classified as worm-hunters, and their venoms are mostly “developed” to prey on submarine worms usually not fatal to mammals. The current study screened six conotoxins from the transcriptome of *C*. *quercinus*, all belonging to α-conotoxins. All these α-conotoxins contain four cysteines, capable of forming the corresponding conotoxins with two disulfide bonds.

Moreover, α-conotoxin has a high affinity with muscle nAChR and is a small peptide toxin rich in disulfides. It is usually 12–20 amino acids in size. These toxins block muscle-type and neuron-type nAChR with high potency and selectivity. For example, GIC selectively inhibits α3β2 nAChR with an IC_50_ of 1.1 nM [42,43]. For nearly 30 years, α-conotoxin has been used as a valuable tool to understand the mechanism of ligand-receptor interaction [44]. The cysteine binding modes of most natural α-conotoxins are Cys^1^-Cys^3^ and Cys^2^-Cys^4^, which usually exhibit the best activity and a “globular” structure [45,46,47,48]. However, there are occasional exceptions with more active “strip” structures (Cys^1^-Cys^4^, Cys^2^-Cys^3^) and “bead” structures (Cys^1^-Cys^2^, Cys^3^-Cys^4^). In particular, Qc1.4, different from other conotoxins in its cysteine binding mode, was selected for the synthesis and activity experiments, whereby an attempt was made to understand the effect of the disulfide bond binding position on activity (Table 1).

In 1966, SPPS was first established by Merrifield et al. [49,50]. Its principle is to immobilize the amino acids at the carboxyl end of peptides on insoluble resins and then synthesize long-chain peptides through amino acid terminal activation, amino acid coupling, and peptide chain extension [51]. Since then, SPPS has become the essential chemical synthesis method for short-chain peptides, overcoming the limitation of step-by-step purification in traditional liquid-phase synthesis [52,53,54].

Conotoxin is the shortest nucleic acid-encoded animal neurotoxin peptide found so far, and it is also the short-chain peptide with the highest cysteine density [55]. Cysteine is one of the sulfur-containing α-amino acids, and its structure contains sulfhydryl groups [56]. Two free sulfhydryl groups can be coupled to form a sulfur–sulfur (disulfide) covalent bond. The disulfide bond is crucial in maintaining the stability of conotoxin 3D structure [57]. Therefore, the synthesized linear conopeptide has no activity. Only by oxidizing the cysteine of conotoxin into a bond and folding it into the natural conformation can it exhibit any physiological activity.

Forming disulfide bonds of conotoxins containing two cysteines is relatively simple, and the free sulfhydryl group can be folded into bonds in one step. However, most conotoxins contain multiple pairs of disulfide bonds. In theory, there are many disulfide bond isomers, and usually, only one cysteine binding mode has biological activity. Step-by-step oxidation is required to synthesize conotoxins with good activity and make cysteine fix-point bonding. The two-step iodine oxidation method improved by Clarence T is highly efficient [58]. Also, as an oxidant, I_2_ can assist dilute hydrochloric acid to promptly remove the Acm protective group in acetic acid solutions, making it more easily oxidized and folded. However, the preliminary experiment found that most linear conopeptides had good solubility in pure water. When exposed to methanol, their solubility sharply decreases, which is necessary for large-scale use in iodine oxidation and is unfavorable for subsequent oxidative folding. The iodine oxidation method was re-optimized (to replace methanol) to improve the poor solubility of hydrophilic peptides in methanol while maintaining the material ratio of Clarence T’s two-step iodine oxidation method.

Based on the insecticidal activity and cytotoxicity data, conotoxins Qc1.15, Qc1.18, Qc-009, and Qc-039 with the first amino acid aspartic acid (D) were consistently outstanding (from the perspective of conotoxin sequence). At the same time, Qc1.12 and Qc1.4 with glutamine (Q) showed inferior water solubility and activity. In addition, Qc1.4 with the cysteine linkage “abnormal” still had specific activity but was slightly lower than other conotoxins.

Amphiphilicity is often considered a key factor affecting the biological activity of peptides [59,60]. It is considered that amphiphilic structures may allow peptides to cross or insert into phospholipid bilayers [61]. In this work, Qc1.15 and Qc1.18 conotoxins showed favorable insecticidal activity both in vivo and in vitro, but according to electrical potential figures, these two conotoxins did not show significant amphiphilicity (Figure 6). Therefore, we speculate that amphiphilicity may not necessarily be related to conotoxins’ insecticidal activity.

Due to their central role in insect neurotransmission, nAChR is an essential molecular target for neurotoxic insecticides [62,63,64]. Molecular docking analysis indicated that Qc1.15 and Qc1.18 conotoxins can interact with the nAChR channels of insects, being the most likely to be responsible for their insecticidal activity (Figure 7). Both amino acid residues of Qc1.15 and Qc1.18 could form hydrogen bonds with Tyr-112 and Lys-116 of nAChR, respectively, suggesting that these two sites on nAChR are crucial. We noticed that Qc1.15 has five more hydrogen bonding interactions with nAChR than Qc1.18. Electric potential analysis indicated that Qc1.15 and Qc1.18 have acidic residues Asp-1 and Asp-15 (Figure 6), but those of Qc1.18 do not form hydrogen bonds with nAChR (Figure 7D). It is speculated that this is due to the inconsistent spatial structure distribution between these two acidic residues and the possible impact of the basic residue His-13 in Qc1.15 (Figure 6). Compared with Qc1.18, the other four conotoxins (Qc-039, Qc-009, Qc1.4, and Qc1.12) all lack the Ser-6 residue (Table 1), essential for hydrogen bonding with Tyr-112 in nAChR (Figure 7D). The cytotoxicity assays and insect bioassay also indicate that the activity of these four conotoxins is relatively low. After comprehensively analyzing this study’s data, we observed that the activity of conotoxin may be related to amino acid composition, peak height, and cysteine binding mode.

**Figure 8:**
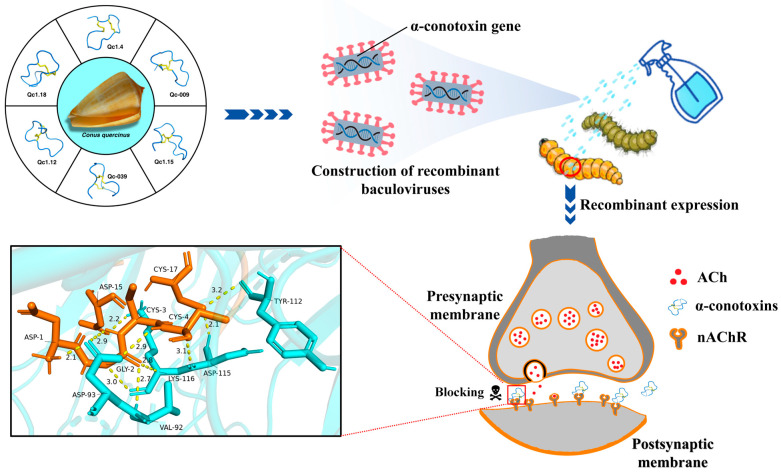
The application prospects and insecticidal mechanism of α-conotoxins.

In the natural ecosystem, insect baculoviruses play a crucial role in maintaining the balance of insect populations, with a pronounced specificity that is usually limited to one or a few closely related insect species. To date, these baculovirus-based biopesticides have been broadly utilized in the agricultural and silvicultural industries of China and Latin America to manage pest infestations [65,66]. However, their application has been somewhat hindered by their slower pace of action and reduced effectiveness against mature insect stages. To address these limitations, genetic engineering has been applied to fortify the lethal potency of baculoviruses, thereby markedly elevating their effectiveness as biocontrol agents [16]. The incorporation of genes encoding for toxins, such as those derived from marine cone snails, arachnids, or other venomous creatures, into the genetic makeup of wild baculoviruses is aimed at boosting their pest-elimination capabilities both in terms of effectiveness and speed. Figure 8 illustrates the application prospects of α-conotoxins, which have been synthesized in this study and exhibit superior insecticidal properties. These toxin genes are integrated into the genome of a baculovirus, enabling their expression within the insect host upon infection. This recombinant approach leverages the baculovirus as a vector to deliver and express the insecticidal toxin, thereby enhancing the efficacy of biological insecticides. This approach has been documented to safeguard a variety of crops, including cotton, poplar, and tobacco, against insect damage [40,67,68].

## 4. Materials and Methods

### 4.1. Screening of α-Conotoxins from C. quercinus

Six α-conotoxin sequences found in the venom ducts of *C*. *quercinus* were screened from over 100 known *C. quercinus* sequences and included in the experiment. Among them, those of Qc1.12, Qc1.15, Qc1.18, and Qc1.4 were obtained by sequencing the transcriptome of *C*. *quercinus* using traditional methods. At the same time, Qc-009 and Qc-039 were selected from the newly discovered 133 conotoxin sequences after re-sequencing the transcriptome of *C*. *quercinus* in the South China Sea using a high-throughput method [13]. Amino acid sequence alignments were performed using ClustalX2.1 and GeneDoc.

### 4.2. Synthesis of Linear Conopeptides

The linear conopeptides were synthesized using the SPPS method [69]. Each conopeptide was assembled on resin from the C-terminus to the N-terminus using a standard Fmoc chemistry strategy. A pair of cysteines in each linear conopeptide (Table 1) was additionally protected with Acm. The crude conopeptides were purified with semi-preparative RP-HPLC (Waters, Taunton, MA, USA) on a Pursuit XRs C18 column (Agilent, Santa Clara, CA, USA) with a linear gradient (5–85%) of Solution B (acetonitrile plus 0.1% TFA). Then, the peptides were characterized by analytical RP-HPLC (Shimadzu, Kyoto, Japan) and TOF-MS (Bruker Daltonics, Bremen, Germany). A linear gradient of solvent B (0.1% TFA in acetonitrile) in solvent A (0.1% TFA in water) was used to increase the percentage of acetonitrile from 5% to 50%. Peptide elution was monitored at 214 nm at a 1 mL/min flow rate. The mass spectra were collected in positive reflector mode after the conopeptide was spotted with the α-cyano-4-hydroxy-cinnamic acid matrix. We used the recommended operation parameters (ion acceleration voltage 20 kV and accumulation time of a single scan 50 s).

### 4.3. Optimization of Oxidative Folding Conditions

The linear conopeptide was oxidized and folded using the two-step iodine oxidation method. By changing the methanol used in different steps, three sets of comparative experiments were completed.

Scheme A: A total of 2 mg linear conopeptide was dissolved in 0.2 mL 50% methanol solution, and 1.8 mL acetic acid was added to dilute the peptide solution. While stirring, 10 mg/mL iodine solution with pure methanol as solvent was added to the peptide solution, resulting in a stable pale yellow color. Continuous stirring for 1 min formed the first disulfide bond. An aliquot of the peptide solution was taken, and the reaction was quenched with L (+) -ascorbic acid (numbered as A^I^). Stirring continued while adding an equal volume of 50 mmol/L hydrochloric acid with 50% methanol solutions to the remaining solution. Then, 0.5 mL of 10 mg/mL iodine solution was added with pure methanol as the solvent. After stirring for 45 min, the second disulfide bond was formed. Then, L (+) -ascorbic acid was added to quench the reaction, and an aliquot of the peptide solution was taken for inspection (A^II^).

Scheme B: A total of 2 mg linear conopeptide was dissolved in 0.2 mL pure water. The remaining steps were consistent with Scheme A.

Scheme C: The 10 mg/mL iodine solution (with pure methanol as the solvent) was replaced with a saturated iodine solution (with pure water as the solvent) and solubilized with potassium iodide. The remaining steps were consistent with Scheme A. After adjusting pH to 5.0–6.0, the six samples were filtered, and the filtrate was collected. The Ultimate 3000 HPLC determined the oxidized conopeptide according to the instrumental method of linear conopeptide.

### 4.4. Oxidative Folding

Using Scheme B (Section 4.3), six linear conopeptides were oxidized and folded in large quantities. The sample was analyzed using the analytical HPLC with the same instrument method as the linear conopeptide and purified repeatedly. The TOF-MS detection methods and conditions were identical to the linear conopeptides. The conotoxin filtrate after the reaction was diluted twice with pure water and purified by P3500 semi-preparative HPLC within 12 h according to the same instrument method as the crude linear conopeptide. After repeated purification and freeze-drying, six kinds of conotoxin freeze-dried powder were obtained.

### 4.5. Cytotoxicity Assays

Here, *S*. *frugiperda* (Sf9) cells were selected to investigate the inhibitory effect of conotoxin on insect cells via the CCK-8 method. Briefly, Sf9 cells in the exponential growth phase were seeded in 96-well plates at 5 × 10^4^ cells/mL concentration. Then, the six conotoxin freeze-dried powders were dissolved in a culture medium, filtered, and added to 96-well plates. The final concentrations of conotoxins in each well were 0.1, 0.5, and 1.0 nM, respectively. After 24 h of culture at 27 °C, the culture medium was discarded, and then CCK-8 reagent was added to each well. After 10 h of culture, the Synergy HTX multi-function microplate detector (Burten Instruments Co., Ltd., Gainesville, FL, USA) detected the optical density (OD) at 450 nm.

### 4.6. Insect Bioassay

*T*. *molitor* of 4–5 instars were selected as experimental insects (body length 2 ± 0.5 cm, average weight 160 mg) and were fed normally before the experiment. Before the experiment, 0.7% saline solution was prepared with normal saline as solvent, and each conotoxin was dissolved to 1, 5, and 10 nM, respectively. Approximately 5 μL was taken using a pointed microsyringe and injected into *T*. *molitor* from the lower abdomen. Each concentration was injected with 9–11 larvae, and three parallel test groups were set up. In addition, three groups were only injected with 0.7% saline solution as a negative control, and three groups were used as blank controls (without any treatment). After that, the *T*. *molitor* was kept in a suitable environment, and deaths within 24 h were observed.

### 4.7. Electrostatic Surface

The 3D structural models of synthesized conotoxins from *C*. *quercinus* were predicted based on amino acid sequences through modeling on the I-TASSER server [70]. PyMol (version 2.6) processed and generated the electric potential figures.

### 4.8. Molecular Docking

To predict the binding mode, Qc1.15 and Qc1.18 conotoxins were selected to dock with the nAChR model of *A*. *pompejana* (PDB Code: 8BX5) [71], using Discovery Studio. The Ramachandran plot of protein 8BX5 is shown in Figure S1. The docking scores of each pose were calculated via the ZDOCK mode, and the complex poses with high scores were refined with the RDOCK model. The Pymol 2.6 downloaded and visualized the binding modes.

### 4.9. Statistical Analysis

Data processing and statistical analyses were performed using GraphPad Prism 8. All data were presented as mean ± SD and compared using one-way ANOVA.

## 5. Conclusions

In this study, we selected six α-conotoxins with possible insecticidal activity from hundreds of conotoxin sequences of *C*. *quercinus*. The corresponding linear conopeptides were gradually synthesized from amino acids using solid-phase peptide synthesis (SPPS). Further, these six linear conopeptides were successfully folded into active conotoxins. Among the α-conotoxins, Qc1.15 and Qc1.18 exhibited high insecticidal activity, excellent in the current conotoxins with proven insecticidal potential. Finally, through homology modeling, electrostatic surface, and molecular docking, the relationship between the structure and biological activity of the α-conotoxins was elucidated, and the insecticidal mechanism of conotoxins was predicted, suggesting that α-conotoxins kill insects by blocking their nAChR. These α-conotoxins with high insecticidal activity that we have screened could be instrumental to construct recombinant baculoviruses and produce efficient and safe biopesticides.

## Figures and Tables

**Table 1:** Alignment of the α-conotoxins from *C. quercinus*.

Name	Conotoxin Sequences	Cysteine Binding Mode	Theoretical Mass (Da)
Qc1.12	QGCCSYPACAVSNPDIC	Cys^1^-Cys^3^ and Cys^2^-Cys^4^	1871.13
Qc1.15	DGCCSDPACAVNHPDIC	Cys^1^-Cys^3^ and Cys^2^-Cys^4^	1860.10
Qc1.18	DGCCSSPSCSVNNPDIC	Cys^1^-Cys^3^ and Cys^2^-Cys^4^	1840.99
Qc1.4	QGCCSDPACAVSNPDIC	Cys^1^-Cys^4^ and Cys^2^-Cys^3^	1823.01
Qc-009	DECCSNPSCAVSNPDIC	Cys^1^-Cys^3^ and Cys^2^-Cys^4^	1897.04
Qc-039	DGCCSNPSCSVNNPDIC	Cys^1^-Cys^3^ and Cys^2^-Cys^4^	1868.09

Note: The same background color highlights amino acid residues with similar properties. Cys^n^ represents the n-th cysteine.

## Data Availability

The original data presented in the study are included in the article; further inquiries can be directed to the corresponding author.

## Supplementary Materials

The following supporting information can be downloaded at: https://www.mdpi.com/article/10.3390/molecules29122846/s1, Table S1: The scores of homology modeling of six α-conotoxins. Figure S1. Ramachandran plot of protein 8BX5.
