# Supplementary material for: Chemical Synthesis and Insecticidal Activity Research Based on α-Conotoxins
Source: Molecules. 2024 Jun 14;29(12):2846. doi: 10.3390/molecules29122846 (PMC11206848; doi:10.3390/molecules29122846)
Supplement: Supplementary file 1 [file molecules-29-02846-s001.zip › molecules-3054815-supplementary.pdf]

Table S1. The scores of homology modeling of six α-conotoxins.

Name	C-score	Exp.TM-Score	Exp.RMSD	No.of decoys	Cluster density
Qc1.4	-0.94	0.60±0.14	2.4±1.8	9022	0.4039
Qc1.12	-0.72	0.62±0.14	2.0±1.6	9206	0.5014
Qc1.15	-0.95	0.59±0.14	2.4±1.8	8618	0.3772
Qc1.18	-0.61	0.64±0.13	1.8±1.5	9567	0.565
Qc-009	-0.91	0.60±0.14	2.3±1.8	8896	0.4405
Qc-039	-0.77	0.62±0.14	2.1±1.7	9434	0.4868

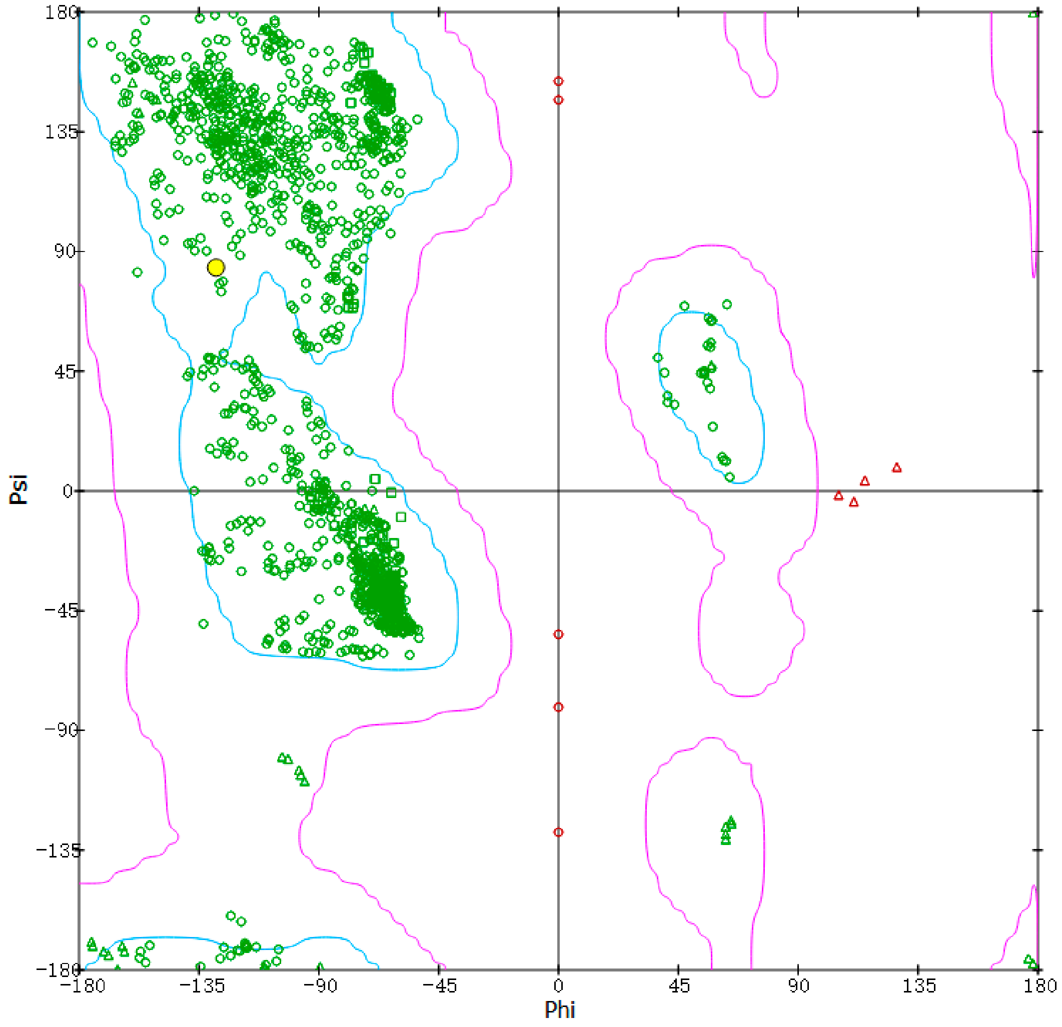

Figure S1. Ramachandran Plot of protein 8BX5.
